# Utilizing journal club to facilitate critical thinking in pre-clinical medical students

**DOI:** 10.5116/ijme.5a46.2214

**Published:** 2018-01-15

**Authors:** Victoria C. Lucia, Stephanie M. Swanberg

**Affiliations:** 1 Department of Biomedical Sciences, Oakland University William Beaumont School of Medicine, USA

**Keywords:** Journal club, critical thinking, pre-clinical medical students

## Introduction

Learning and practicing evidence-based medicine (EBM) is vital for all healthcare professionals throughout their careers and requires expert medical knowledge, effective communication skills to uncover patient values, as well as critical thinking skills in appraisal of the scientific literature.^1^ Integrating EBM training and critical thinking skills into undergraduate medical education programs is required and fully supported by accrediting bodies, such as the Liaison Committee on Medical Education (LCME) and professional organizations.^2^^,^^3^ Specifically, in their 2014 report, Core Entrustable Professional Activities (EPAs) for Entering Residency, the Association of American Medical Colleges (AAMC) named retrieving and applying evidence as a fundamental skill for graduating medical students.^3^ Core EPAs for entering residency is an initiative to define a "...common core set of behaviors that could/should be expected of all [medical school] graduates".^3^

One way to incorporate these fundamental skills into undergraduate medical education is through the use of journal clubs. Journal clubs are already a well-documented instructional method used in residency programs and for continuing medical education.^4^^,^^5^ In that journal clubs focus on critically evaluating current literature and applying it to patient care, they provide the perfect environment for teaching EBM and have been shown to positively impact EBM knowledge and skills.^6^^,^^7^ Effectiveness of these journal clubs has depended on several characteristics including a clear purpose, incentives, and a trained journal club leader.^8^

Despite extensive use at higher medical education levels, use of journal clubs has been minimally reported in the undergraduate pre-clinical medical education literature. Most studies have implemented journal clubs during medical students' clinical years.^9^^,^^10^ The purpose of this article is to describe the successes and challenges of, as well as lessons learned in implementing, structured journal club activities in the pre-clinical years in an effort to introduce critical thinking and application skills.

### Educational activity

In their first year of medical school, medical students participate in two mandatory small group journal club sessions, facilitated by faculty and staff, during a longitudinal prevention and public health course. Journal articles were selected to align with general topics being taught in the greater curriculum and included childhood obesity, use of menthol in cigarettes, and cardiac screening in high school athletes. As part of course assessments, students were required to submit written responses to instructor-written questions one week prior to the discussion session. Requiring a graded written assignment assured that all students thoroughly read and thought critically about the article and associated guidelines or public policies related to the selected topic in order to actively participate in the discussion session. The written assignment also required students to apply general study findings to specific subsets of the population, including identifying barriers and social-determinants of health. Students were divided into discussion groups of five to seven medical students with a faculty or staff facilitator, who was provided with each of their students' written assignments. Discussion sessions were 45 minutes in length. A facilitator guide was also provided to all faculty and staff, but they were instructed to allow the discussion to be guided by student group interest and direction more than an instructor-set agenda.

To assess the impact of the activity and continuously improve it, students and facilitators provided feedback about the journal club activities at the conclusion of the semester. Overall, students found the group discussion to be beneficial in understanding and appreciating other people's points of view, as well as providing a deeper understanding of relevant and current public health issues. Students did not perceive any benefit from completing the written assignment; they suggested that grading be centered on the group discussion rather than the written assignment. However, facilitators believed the completion of the written assignment was instrumental in preparing students for the group discussion. Facilitators also thought that this structured activity encourages students to integrate concepts from multiple courses and to think critically about relevant health care issues during the group discussion.

### Lessons learned

Through this experience over the past three academic years, we have learned some important lessons for implementing and maintaining such an activity. Given that the literature is always changing, it is important to remain flexible both in the selection of articles and the structure of written assignments and discussion. Soliciting feedback for improvement from both students and facilitators, as well as implementing appropriate changes over time, is imperative. In addition, as has been found in previous research with residents, mandatory attendance, a clear purpose, and trained facilitators also proved to be important factors in the success of these journal club activities.^8^ Finally, these journal clubs also provide an introduction to the critical appraisal skills that students encounter again during the optional journal club sessions held in second year, in their two-week dedicated EBM course in the second year, and throughout their two clinical years, particularly the Internal Medicine clerkship in the third year. This creates a vertically-integrated curricular thread that reinforces important knowledge and skills throughout the curriculum.

## Conclusions

Critical appraisal of scientific literature and the ability to appropriately apply findings to patients is an essential skill for the practice of EBM. Our experience demonstrates that, despite some challenges, journal clubs can be successfully implemented to introduce first-year medical students to the critical thinking and real-world application skills expected at graduation and needed throughout their careers. While students found the discussion aspect of the journal club more interesting and helpful to their training, facilitators found value in both the preparatory written assignment and group discussion in preparing first-year students for future, formal pre-clinical training in EBM and practice in the clinical years. This simple, but effective, educational activity could easily be implemented at other institutions early in their medical education curricula as a learning and assessment tool.

### Acknowledgements

The authors would like to thank Leon Pedell, MD for his commitment to medical education and his guidance in identifying timely and content-appropriate articles for this educational activity.

### Conflict of Interest

The authors declare that they have no conflict of interest.
